# Positive Effects of Openness on Cognitive Aging in Middle-Aged and Older Adults: A 13-Year Longitudinal Study

**DOI:** 10.3390/ijerph16122072

**Published:** 2019-06-12

**Authors:** Yukiko Nishita, Chikako Tange, Makiko Tomida, Rei Otsuka, Fujiko Ando, Hiroshi Shimokata

**Affiliations:** 1 Department of Epidemiology of Aging, National Center for Geriatrics and Gerontology, Aichi 474-8511, Japan; 2 Section of NILS-LSA, National Center for Geriatrics and Gerontology, Aichi 474-8511, Japan; tange@ncgg.go.jp (C.T.); tomida@ncgg.go.jp (M.T.); otsuka@ncgg.go.jp (R.O.); fujikoa@asu.aasa.ac.jp (F.A.); simokata@nuas.ac.jp (H.S.); 3 Faculty of Health and Medical Sciences, Aichi Shukutoku University, Aichi 480-1197, Japan; 4 Graduate School of Nutritional Sciences, Nagoya University of Arts and Sciences, Aichi 470-0196, Japan

**Keywords:** cognitive aging, openness, community-dwellers

## Abstract

The relationship between openness (a psychological trait of curiosity) and a cognitive change was examined in middle-aged and older adults. Participants were 2214 men and women (baseline age range: 40 to 81 years). They were tested up to seven times over approximately 13 years. Openness at the baseline was assessed by the NEO Five-Factor Inventory. Cognitive abilities were assessed at each examination using the Wechsler adult intelligence scale-revised short form, which includes information, similarities, picture completion, and digit symbol subscales. General linear mixed models comprised fixed effects of openness, age at the baseline, follow-up time, their interactions, and the covariates. The results indicated that the main effects of openness were significant for all scores. Moreover, the interaction term openness × age × time was significant for the information and similarities test scores, indicating that changes in the information and similarities scores differed depending on the level of openness and baseline age. The estimated trajectory indicated that the differences in slopes between participants with high and low openness were significant after 60 years of age for the information, and after 65 years of age for the similarities scores. It is concluded that openness has a protective effect on the decline in general knowledge and logical abstract thinking in old age.

## 1. Introduction

The population of the world is aging dramatically. The life expectancies of Japanese people were 58.0 years for men and 61.5 years for women in 1950. In comparison, life expectancy was 81.1 years for men and 87.3 years for women, and people aged 65 years and over comprised 27.7 % of the total population in 2017 [1]. A longer and healthier life has become an increasing concern for individuals and our society.

Cognitive abilities are important contributors to living better for older people, regardless of dementia. Cognitive abilities are associated with physical functions [2,3] and the ability to solve everyday problems [4,5,6]. Furthermore, cognitive abilities affect physical health and longevity through the understanding of psychosomatic conditions and their management [7,8]. Therefore, there is an urgent social and academic need to identify factors contributing to individual differences in cognitive abilities. The current study focused on openness, a psychological trait, as a factor influencing cognitive aging.

Openness is one dimension of the Big Five personality traits, and it is defined as the degree of openness to various new experience [9,10]. Individuals with high openness have more curiosity and interest in their inner and outer worlds and enjoy trying new things [9]. Openness has been consistently associated with better cognitive abilities when measured concurrently [11,12,13]. However, we have not yet fully understood how openness is longitudinally associated with cognitive aging. Does openness affect not only the level but also the change in cognitive abilities? Do the effects of openness on cognitive abilities differ by age or cognitive dimension?

The purpose of this study was to investigate the relationship between openness and cognitive change in community-dwelling people. Significant aspects of this study included the following: (1) The participants were followed for 13 years; (2) the participants spanned a wide range of ages, from middle to old age; and (3) cognitive abilities were assessed by neuropsychological tests, the Wechsler adult intelligence scales-revised short forms (WAIS-R-SF) [14], which is an internationally used tool for assessing multiple cognitive abilities. The WAIS-R-SF consists of four standardized subtests that are designed to fall into normal distributions for adults of all ages and are extracted to reflect the overall intelligence of an individual. Therefore, it is suitable for evaluating individual differences of multiple cognitive dimensions in community-dwelling adults with a wide age range.

## 2. Methods

### 2.1. Participants

The data for this study were collected as a part of the National Institute for Longevity Sciences, Longitudinal Study of Aging (NILS-LSA). The NILS-LSA is a Japanese population-based prospective cohort study of normal aging and age-related diseases. The participants were age- and sex-stratified random samples selected from the neighborhood of the institute. Wave 1 of the NILS-LSA was conducted from 1997 to 2000 and included 2267 participants (1139 men and 1128 women; age range: 40–79 years). Participants were followed up almost every two years: Wave 2 (2000–2002), Wave 3 (2002–2004), Wave 4 (2004–2006), Wave 5 (2006–2008), Wave 6 (2008–2010), Wave 7 (2010–2012), and Wave 8 (2013–2016). If any participants were unable to attend the follow-up investigations, we randomly recruited new, age- and sex-matched participants from the same residential area. Moreover, new participants with ages in their 40s were also recruited at every wave. The protocol of the study was approved by the Committee on Ethics of Human Research of the National Center for Geriatrics and Gerontology (No. 899-3). Written, informed consent was obtained from all participants. Details of the NILS-LSA have been reported elsewhere [15].

The baseline for this study was the Wave 2 examination (*n* = 2259) because data on openness (explained below) in the NILS-LSA were available at Wave 2. Individuals were excluded if they (a) had a history of dementia at the baseline (*n* = 4); (b) had missing data on all cognitive abilities at the baseline (the details are explained below; *n* = 7); or (c) had missing data on openness and control variables at the baseline (details are explained below; *n* = 34). Based on these criteria, 2214 individuals were included in this study (women: 49.01%; mean age at baseline = 59.69 years, SD = 11.35 years; age range = 40–81 years). The mean baseline MMSE (mini-mental state examination) [16] score of the participants aged 60 years and above that was available in NILS-LSA was 27.87 (SD = 1.96). 

Table 1 shows details of the follow-up participation, from Wave 3 to Wave 8. The mean follow-up duration time from baseline to final assessment was 10.44 (SD = 4.01) years. The average number of measurements was 5.08 (SD = 2.17) per participant. A total of 11,263 cumulative observations of the 2214 participants were analyzed. The participation rates in all the follow-up examinations had no significant association with sex (data not shown).

### 2.2. Measures

#### 2.2.1. Cognitive Abilities (Baseline and all Follow-up Surveys)

Cognitive abilities were assessed by the Japanese version of the Wechsler adult intelligence scale-revised short form (WAIS-R-SF) [14]. Trained testers, who were clinical psychologists or psychology graduate students, individually administered the test to each participant. The WAIS-R-SF consists of the following tests: Information, similarities, picture completion, and digit symbol. The information test assesses general knowledge by asking participants general knowledge questions covering people, places, and events (29 items, possible range 0–29). The similarities test assesses logical abstract thinking, by asking participants to state how two things are similar (14 items, possible range 0–28). The picture completion test assesses visual perception and long-term visual memory, by asking participants to spot the missing elements in a series of drawings (21 items, possible range 0–21). The digit symbol test assesses processing speed by asking participants to write as many symbols as possible that correspond to a given number in 90 seconds (possible range 0–93). Higher scores in all the tests indicate better performance.

#### 2.2.2. Openness (Baseline)

Openness was assessed by a subscale of the Japanese version of the NEO Five-Factor Inventory (NEO-FFI), which is a self-administered questionnaire [10]. The participants were asked to answer it prior to the survey day and bring the questionnaire to the survey. The openness subscale of NEO-FFI includes 12 items, with scores ranging from 0 to 48, such that higher scores indicate higher levels of openness. Cronbach’s alpha coefficient of this sample was 0.61, which is relatively low. However, NEO-FFI is the most common, internationally used tool for assessing personality. Previous studies have reported that the internal consistency of the Japanese openness subscale tends to be relatively low [17]. Therefore, we adopted this scale as an indicator of openness.

#### 2.2.3. Covariates (Baseline)

At the baseline, data on education level (years), marital status (unmarried, married), occupation (no occupation, having an occupation), current smoking (nonsmoker, smoker), and past and present illnesses (stroke, hypertension, heart disease, and diabetes: None, having past, or present illnesses) were collected by means of a self-administered questionnaire. Follow-up years was calculated as the time from baseline to each participation day.

### 2.3. Statistical Analysis

General linear mixed models were used to evaluate the effects of openness on cognitive changes over a 13-year period. General linear mixed models can handle missing data more appropriately than traditional regression analysis and repeated measures analysis. The correlations between the repeated measures are adequately accounted for by the variance-covariance structure of random effects. Further information on the application of general linear mixed models to repeated measures data is published elsewhere [18,19]. Many recent studies have used the general linear mixed model for analyzing cognitive change [20,21,22]. 

The model used in this study included fixed terms for the intercept (baseline performance with value zero on all predictors), age (at the baseline), openness (at the baseline), time (time in years since the baseline, i.e., slope), and the interaction terms focusing on time; age × time, openness × time, and age × openness × time. Sex, education level, marital status, occupation, current smoking, past and present illnesses, and practice effects (prior exposure to the cognitive tests) were included as covariates. The terms of primary interest in this study were the openness × time and age × openness × time interactions. The openness × time interaction was expected to reflect whether the level of openness affected cognitive change (slope). The age × openness × time interaction was expected to indicate whether the relationship between openness and cognitive change (slope) varied according to the baseline age. Additionally, the models included random intercepts and time effects, which captured subject-specific deviations from the intercept and slope of the population mean.

Openness, baseline age, and all covariates were centered at the sample mean, so that the estimated parameters indicated the sample-average effects. 

Prior to the analyses, we examined non-linear models including time-squared terms and their interactions (i.e., the models including fixed terms for the intercept, age, openness, time, time × time, age × time, age × time × time, openness × time, openness × time × time, openness × age × time, openness × age × time × time, and covariates). The results indicated that none of the time-squared terms were significant for any of the cognitive ability scores. Therefore, we adopted linear models excluding the time-squared terms. Then, we examined the models including the interactions between time and covariates. However, most interaction terms of time and covariates were not significant (except for the significant interaction of time and education for the digit symbol score). The results of the models including the interaction of time and covariates were nearly identical to the results of the models excluding them. Therefore, we adopted the simpler models that only included the main effects of covariates.

Statistical analyses were conducted using the SAS System version 9.3 (SAS Institute, Cary, NC, USA). A *p*-value of <0.05 was considered to be statistically significant.

## 3. Results

### 3.1. Baseline Characteristics

Table 2 shows the baseline characteristics of the participants, including age, sex, education level, marital status, occupation, current smoking, past and present illnesses, cognitive abilities scores, and openness score. Average years of education were 11.9 (SD = 2.8) years. There were 1929 (87.1%) married people, 1253 (56.6%) workers, and 455 (20.6%) smokers. The distribution of past and present illnesses was as follows: 72 participants (3.3%) had strokes, 583 (26.3%) participants had hypertension, 118 participants (5.3%) had heart disease, and 179 participants (8.1%) had diabetes. Their average cognitive abilities scores were 15.2 (SD = 5.6) for information, 13.9 (SD = 5.5) for similarities, 11.4 (SD = 3.6) for picture completion, and 53.3 (SD = 16.0) for digit symbol. Moreover, their average openness score was 25.8 (SD = 4.3). The cognitive abilities of the participants in this study were higher and their openness score was a little lower compared to the reference data of the WAIS-R-SF [14] and the NEO-FFI [10] manuals (data not shown).

### 3.2. Openness and Cognitive Changes

**Figure 1:**
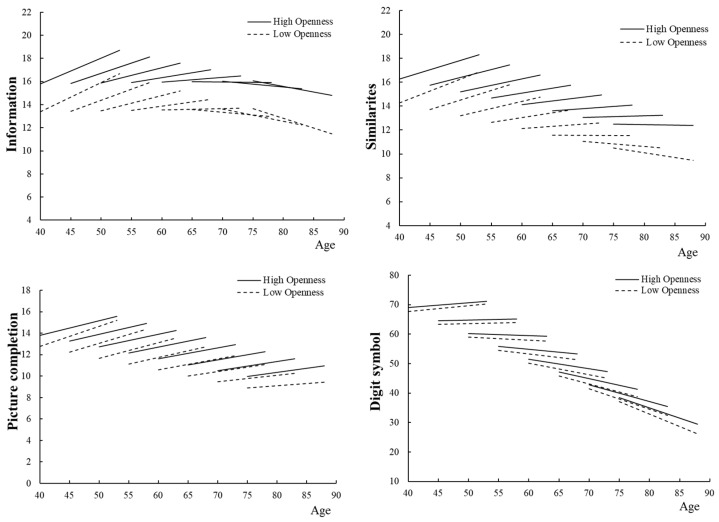
Model-predicted 13-year changes in cognitive abilities by openness and baseline age. The solid lines are estimated for individuals with a high openness level (openness score = 30.12); the dashed lines are estimates for individuals with a low openness level (openness score = 21.46). Higher scores indicate a better performance. Possible score for the information is 0–29; similarities 0–28; picture completion 0–21; and digit symbol 0–93. The models controlled for sex, education level, marital status, occupation, smoking, past and present illness, and practice effects.

Table 3 shows estimates of the general linear mixed models. Figure 1 shows 13-year changes in cognitive abilities predicted by the model, according to substituting baseline age (five-year-separated) and openness level (mean ± 1 SD; low openness = 21.46, high openness = 30.12). On average, the cognitive scores except for the information score declined with age (the main effects of age; information *p* = 0.533, similarities, picture completion, and digit symbol *p* < 0.001). The average cognitive changes through the follow-up time for digit symbol were negative whereas changes in the other scores were positive (all subtests *p* < 0.001). Moreover, the variance of the random intercept and slope of each cognitive ability was significant (all *p* < 0.001), indicating that there were inter-individual differences in baseline scores and the changes.

The main effects of openness were significant for the all the cognitive domains (information, similarities, and picture completion *p* < 0.001; digit symbol *p* = 0.002), indicating that information, similarities, picture completion, and digit symbol scores were related to the overall level of openness. In addition, the interaction effects of openness × age × time, which were our primary interest, were significant for the information score and the similarities score (*p* = 0.022; *p* = 0.009), indicating that changes in the information score and the similarities score differed according to the level of openness and baseline age. As shown in Figure 1, predicted trajectories of the information and similarities scores obtained by substituting baseline age and openness level, indicated that differences in slopes between those with high and low openness were significant after 60 years of age for information and after 65 years of age for similarities. The information score started to decline significantly from age 63 in participants with low openness, whereas it only started to decline from age 69 in participants with high openness. The similarities score started to decline significantly from age 69 in participants with low openness, whereas it did not decline until age 79, which was the age of the oldest participant in this study, with high openness. On the other hand, the terms of primary interest in this study, openness × time and openness × age × time were not significant for the picture completion or the digit symbol scores.

The variance of the random intercept and slope was statistically significant for all cognitive abilities (*p* < 0.001), which was indicative of individual differences at baseline and in change over time for these abilities. The covariance between the random intercept and slope was significant for similarities and picture completion (*p* = 0.018; *p* < 0.001), indicating that baseline scores were associated with the rate of change over time.

## 4. Discussion

The current study investigated the longitudinal associations between openness and cognitive changes over a 13-year follow-up period among community-dwelling middle-aged and older people. 

Large-scale longitudinal studies, such as the Seattle Longitudinal Study [23] and the Berlin Longitudinal Study [24] have indicated that cognitive aging differs for different domains. As shown in Figure 1, this study also showed that mean longitudinal changes in cognitive functions during 13 years were very different for different aspects of cognitive functions. Moreover, the main results indicated that openness affected cognitive abilities, and these effects were different depending on baseline age and cognitive domain.

Openness is an intellect dimension of the five-factor model of personality [9,10]. Openness, relative to other personality factors, has been more strongly correlated with cognitive abilities [11,12,13]. The results of the current study, which corroborate previous studies [11,12,13], suggest that openness and cognitive abilities are significantly correlated in middle-aged and older people. Moreover, there were especially notable and significant interactions among age, openness, and follow-up years that affected the information and similarities test score, such that openness affected longitudinal changes in the information and similarities score during the subsequent 13 years. In addition, these effects were more pronounced with age increasing.

The information and similarities test are verbal tests of the WAIS-R-SF that reflect crystallized intelligence, which is accumulated through life experiences and education [25,26]. The results of this study indicated that when openness was high, crystallized intelligence was better maintained to an older age (up to 69 years of age for information; and up to 79 years of age for similarities), whereas when openness was low, crystallized intelligence started to decline at a relatively younger age of 63 years for information and 69 years for similarities. These findings suggest that openness is an important factor related to individual differences in changes of crystallized intelligence, especially in older people. That is, a personality with openness to new experiences and a high degree of curiosity might be more useful for maintaining crystallized intelligence, which includes general knowledge and logical abstract thinking. Crystallized intelligence could compensate for the decline in other cognitive abilities such as fluid abilities or processing speed [27]. Moreover, crystallized intelligence might have the function of cognitive reserve that could be useful for preventing dementia [28]. Due to the importance of crystallized intelligence, the results of this study indicating positive effects of openness on crystallized intelligence might suggest practical methods of maintaining overall cognitive functions and preventing dementia.

Why were these longitudinal effects observed in only older people? Most older people tend to be retired, and their children tend to have become independent. People could have more freedom and more choice, as well as more opportunities to pursue personal interests [29]. Van Solinge and Henkens [30] regarded openness as one of the resources that provide support during significant challenges, which subsequently affects successful aging. In addition, Stephan [11] suggested that openness includes intellectual flexibility and that older people with a high degree of openness could flexibly address various changes faced in old age. Thus, the flexibility that is associated with openness might explain individual differences in the change of cognitive abilities in older people.

There were significant cross-sectional associations of the picture completion and digit symbol test scores with openness, although the interaction between openness and the follow-up years was not significant. These findings suggest that middle-aged and older people with curiosity and tendencies to enjoy new experiences tend to have a better visual perception, long-term visual memory, and higher processing speed. No studies have examined the relationships between openness and picture completion test performance. Moreover, the results of previous studies on the relationships between openness and digit symbol test performance have been mixed. Specific previous studies have indicated no cross-sectional correlations between processing speed and openness [31], whereas other studies have suggested significant correlations [23,32,33]. Therefore, it is significant that the current study, which included a large number of middle-aged and older community dwellers, found that the picture completion and digit symbol test score were correlated with openness.

Picture completion and digit symbol tests are performance tests of the Wechsler adult intelligence scale-revised that reflect fluid intelligence [25,26]. Fluid intelligence is the ability to solve problems when adapting to new learning [26] and is a significant resource for dealing effectively with new experiences [32]. Therefore, it might be possible to assume reverse causal relationships between fluid intelligence and a longitudinal change of openness, rather than between openness and longitudinal changes of fluid intelligence. We suggest that future studies need to investigate the causal relationships between openness and cognitive abilities.

This study has some limitations. First, participants were a stratified random sample of community-dwelling middle-aged and older people; however, they were relatively physically or mentally healthy people who could repeatedly travel to participate in the study at our institution in Japan. Therefore, our findings cannot be extrapolated to sick or disabled people or people all over the world. Second, this study used the NEO-FFI to investigate how openness at the baseline affected cognitive decline. However, multiple facets of openness have been identified [9]. More research using other personality scales, such as the Revised NEO Personality Inventory [9], is needed to examine whether or which facets of openness prevent cognitive decline. Third, in this study, we only evaluated personality at the baseline. However, it is known that personality could change through experiencing life events as well as social and environmental transitions of adulthood, and later [34]. Therefore, it is suggested that future research investigate how changes in personality influence cognitive changes and vice versa. Finally, it is possible that the results of this study depended on the number of follow-up years. For example, the models of picture completion and digit symbol scores did not show significant interactions between openness and time. However, these results might have been different if longer follow-up periods had been included. As a result, we suggest that further studies with extended follow-up periods are included in future studies.

## 5. Conclusions

Openness had a protective effect on the decline in general knowledge and logical abstract thinking in old age.

## Figures and Tables

**Table 1:** Information on follow-up participation.

	Participants, *n*	Follow-up Years from Baseline, *mean* (*SD*)	
Wave 2 (Baseline)	2214	0.00	
Wave 3	1872	2.06	(0.09)
Wave 4	1708	4.18	(0.26)
Wave 5	1594	6.23	(0.35)
Wave 6	1458	8.22	(0.38)
Wave 7	1335	10.20	(0.42)
Wave 8	1082	13.53	(0.45)

**Table 2:** Participant characteristics at baseline.

Age at baseline, year, *mean* (*SD*)	59.69	(11.35)
Sex, women, *n* (*%)*	1085	(49.01)
Education level, year, *mean* (*SD*)	11.87	(2.78)
Marital status, married, *n* (*%*)	1929	(87.13)
Occupation, having occupation, *n* (*%*)	1253	(56.59)
Current smoking, smoker, *n* (*%*)	455	(20.55)
Past and present illness, *n* (*%*)		
Stroke	72	(3.25)
Hypertension	583	(26.33)
Heart disease	118	(5.33)
Diabetes	179	( 8.08)
Cognitive abilities score, *mean* (*SD*)		
Information	15.16	(5.58)
Similarities	13.85	(5.52)
Picture Completion	11.38	(3.56)
Digit Symbol	53.32	(15.95)
Openness score, *mean* (*SD*)	25.79	(4.33)

**Table 3:** General linear mixed model parameter estimates for cognitive abilities.

	Information			Similarities			Picture Completion			Digit Symbol Score		
	Estimate	SE	*p*-value	Estimate	SE	*p*-value	Estimate	SE	*p*-value	Estimate	SE	*p*-value
*Fix efffects*												
Intercept	15.158	0.097	<0.001	13.846	0.091	<0.001	11.376	0.062	<0.001	53.280	0.208	<0.001
Openness (at baseline)	0.260	0.021	<0.001	0.216	0.019	<0.001	0.112	0.013	<0.001	0.140	0.046	0.002
Age (at baseline)	0.007	0.011	0.533	–0.108	0.010	<0.001	–0.111	0.007	<0.001	–0.874	0.024	<0.001
Time (follow–up years)	0.030	0.007	<0.001	0.052	0.008	<0.001	0.103	0.006	<0.001	–0.351	0.016	<0.001
Openness × Time	0.003	0.001	0.054	0.002	0.001	0.134	0.000	0.001	0.939	0.008	0.007	0.074
Age × Time	–0.011	0.001	<0.001	–0.006	0.001	<0.001	–0.003	0.000	<0.001	–0.027	0.001	<0.001
Openness × Age × Time	0.0003	0.0001	0.022	0.0004	0.0001	0.009	0.0003	0.0002	0.059	0.0006	0.0003	0.053
Edcation level	0.896	0.040	<0.001	0.725	0.035	<0.001	0.197	0.023	<0.001	1.334	0.086	<0.001
Sex	–2.028	0.212	<0.001	–0.541	0.185	0.004	–0.953	0.122	<0.001	1.683	0.457	0.000
Current smoking	–0.854	0.244	0.001	–0.797	0.213	0.000	–0.332	0.141	0.018	–2.238	0.527	<0.001
Stroke	–0.666	0.536	0.214	–0.702	0.475	0.140	–0.177	0.316	0.577	–3.079	1.154	0.008
Hypertension	–0.176	0.222	0.428	–0.109	0.194	0.575	–0.162	0.129	0.208	–0.566	0.479	0.237
Heart disease	0.238	0.420	0.570	0.113	0.371	0.761	–0.081	0.246	0.743	0.533	0.904	0.555
Diabetes	–0.090	0.343	0.793	–0.264	0.302	0.383	–0.223	0.200	0.266	–1.855	0.742	0.012
Marital status	–0.003	0.288	0.992	0.046	0.253	0.856	0.270	0.167	0.106	0.941	0.621	0.129
Occupation	–0.344	0.226	0.128	0.083	0.197	0.674	–0.085	0.130	0.513	0.240	0.487	0.623
Practice effect	0.216	0.061	<0.001	–0.026	0.075	0.726	0.398	0.054	<0.001	1.908	0.124	<0.001
*Random efffects*												
Variance of intercept	17.066	0.574	<0.001	12.819	0.483	<0.001	5.663	0.221	<0.001	80.848	2.690	<0.001
Variance of slope	0.024	0.002	<0.001	0.013	0.003	<0.001	0.008	0.001	<0.001	0.122	0.010	<0.001
Covariance intercept and slope	0.014	0.026	0.585	–0.065	0.028	0.018	–0.053	0.014	<0.001	–0.152	0.123	0.216
Residual	3.599	0.059	<0.001	5.664	0.093	<0.001	2.888	0.048	<0.001	14.861	0.247	<0.001

Higher scores indicate better performance. Possible score for the Information is 0–29; Similarities 0–28; Picture Completion 0–21; Digit Symbol 0–93. The baseline age, openness and all covariates were centered at the sample mean.
